# Reliability optimization of process parameters for marine diesel engine block hole system machining using improved PSO

**DOI:** 10.1038/s41598-021-01567-x

**Published:** 2021-11-09

**Authors:** Honggen Zhou, Weibin Yang, Li Sun, Xuwen Jing, Guochao Li, Liping Cao

**Affiliations:** 1 School of Mechanical Engineering, Jiangsu University of Science and Technology, Zhenjiang, 212000 China; 2 Shaanxi Diesel Engine Heavy Industry Company Limited, Xingping, 713100 China

**Keywords:** Mechanical engineering, Engineering

## Abstract

The processing quality of the block hole system affects the working performance of the marine diesel engine block directly. Choosing an appropriate combination of process parameters is a prerequisite to improving the accuracy of the block hole system. Uncertain fluctuations of process parameters during the machining process would affect the process reliability of the block hole system, resulting in an ultra-poor accuracy. For this reason, the RBF method is used to establish the relationship between the verticality of the cylinder hole and process parameters, including cutting speed, depth of cut, and feed rate. The minimum cylinder hole verticality is taken as the goal and the process reliability constraints of the cylinder hole are set based on Monte Carlo, a reliability optimization model of processing parameters for cylinder hole is established in this paper. Meanwhile, an improved particle swarm algorithm was designed to solve the model, and eventually, the global optimal combination of process parameters for the cylinder hole processing of the diesel engine block in the reliability stable region was obtained.

## Introduction

The block, which is a large thin-walled structure component, is one of the most important parts of the marine diesel engine. The processing of the block holes is a key process in block processing and their quality would affect the performance, accuracy, and life of the block directly. The process parameters, such as the cutting speed, the feed rate, and soon, play a vital role to ensure the accuracy of holes machining. Therefore, optimizing the process parameters according to the processing requirements of the block hole system is of great significance to improve the processing quality of the diesel engine block.

It is a common method to optimize the process parameters by exploring the relationship between the process parameters and the optimization objective through the experimental design. Awale et al.^1^ researched the influence of high-speed turning parameters on the surface roughness of harden AISI S7 tool steel by signal-to-noise ratio analysis method, and the results showed that higher cutting speed and lower feed rate can significantly improve the surface quality of hardening AISI S7 tool steel. Campatelli et al.^2^ conducted milling experiments on AISI 1050 carbon steel workpiece by NMV1500DC five-axis milling machine, and the process parameters with the lowest environmental footprint were obtained based on the response surface method, which are higher cutting speed, feed rate, and chip section. Pervaiz et al.^3^ used Taguchi analysis to design inclined drilling tests of Inconel 718 under different process parameters, and the test results showed that feed rate has the greatest impact on cutting force, while spindle speed has the greatest impact on cutting power and cutting temperature parameter.

The intelligent optimization algorithm has also been effectively applied to the optimization of process parameters. Genetic algorithm^4,5^ and simulated annealing algorithm^6,7^ take the output of the prediction model as the fitness function. Based on the rule algorithm, the optimal combination of process parameters can be solved through repeated iterations to meet the required fitness requirements. Particle swarm optimization algorithm^8,9^ has become a widely used algorithm in process parameter optimization by its relative real value particle structure and faster iteration speed. However, with the progress of intelligent control optimization theory, the defects of standard intelligent optimization algorithms are gradually exposed. For example, the convergence speed is slow and the precision is low under multiple constraints for genetic algorithm^10^, and the optimization result of particle swarm optimization algorithm is easy to fall into the local optimal solution because of its randomness^11^. Therefore, the improvement of intelligent optimization algorithms has become a way to obtain more accurate optimal solutions of process parameter combination. Chu et al.^12^ proposed a hybrid Taguchi genetic algorithm to solve the optimal combination of lathe process parameters with the optimization objectives of material removal rate and surface roughness. The experiment results showed that the hybrid Taguchi genetic algorithm has better convergence and robustness than the traditional GA algorithm. Tan et al.^13^ combined a new chaotic search strategy with particle swarm optimization to solve constrained programming problems, and experiments indicated that the hybrid algorithm not only has better convergence than other chaotic search algorithms, but also has better performance in dealing with high-dimensional problems. An improved artificial bee colony algorithm was presented to solve the constrained optimization problem^14^, which used a fixed proportion of direct comparison rules to select individuals and introduces the optimal solution information in the reconnaissance bee stage. Finally, based on the standard test function, it was proved that the improved artificial bee colony algorithm works better than the basic artificial bee colony algorithm for most test functions. There are many types of research on the optimization of process parameters, but few people pay attention to the influence of the uncertainty fluctuation of process parameters on the processing quality. Tian^15^ provides a method to establish and evaluate the process reliability model of the diesel engine block, but this method can’t control the process reliability of the block from the process design itself. Therefore, based on his research, this paper integrates the reliability theory in the process of optimization, and the reliability stability region of the block is selected based on the Monte Carlo method. The minimum verticality of the diesel engine block cylinder hole is taken as the optimization objective, and the Hooke-Jeeves algorithm is combined with the particle swarm optimization algorithm to solve the established reliability optimization model. The optimal process parameters of the diesel engine block hole system obtained according to the above method can not only satisfy the high machining accuracy of the block hole system, but also ensure the reliability of the diesel engine block. To a certain extent, the method in this paper has made a contribution to improving the machining process technology and reliability of the diesel engine for marine diesel engine manufacturers. In addition, the proposed method in this paper can provide some reference and new ideas for the improvement of the manufacturing process of products with small samples.

## Establishment of approximate model

### Design variables and optimization objectives

**Figure 1:**
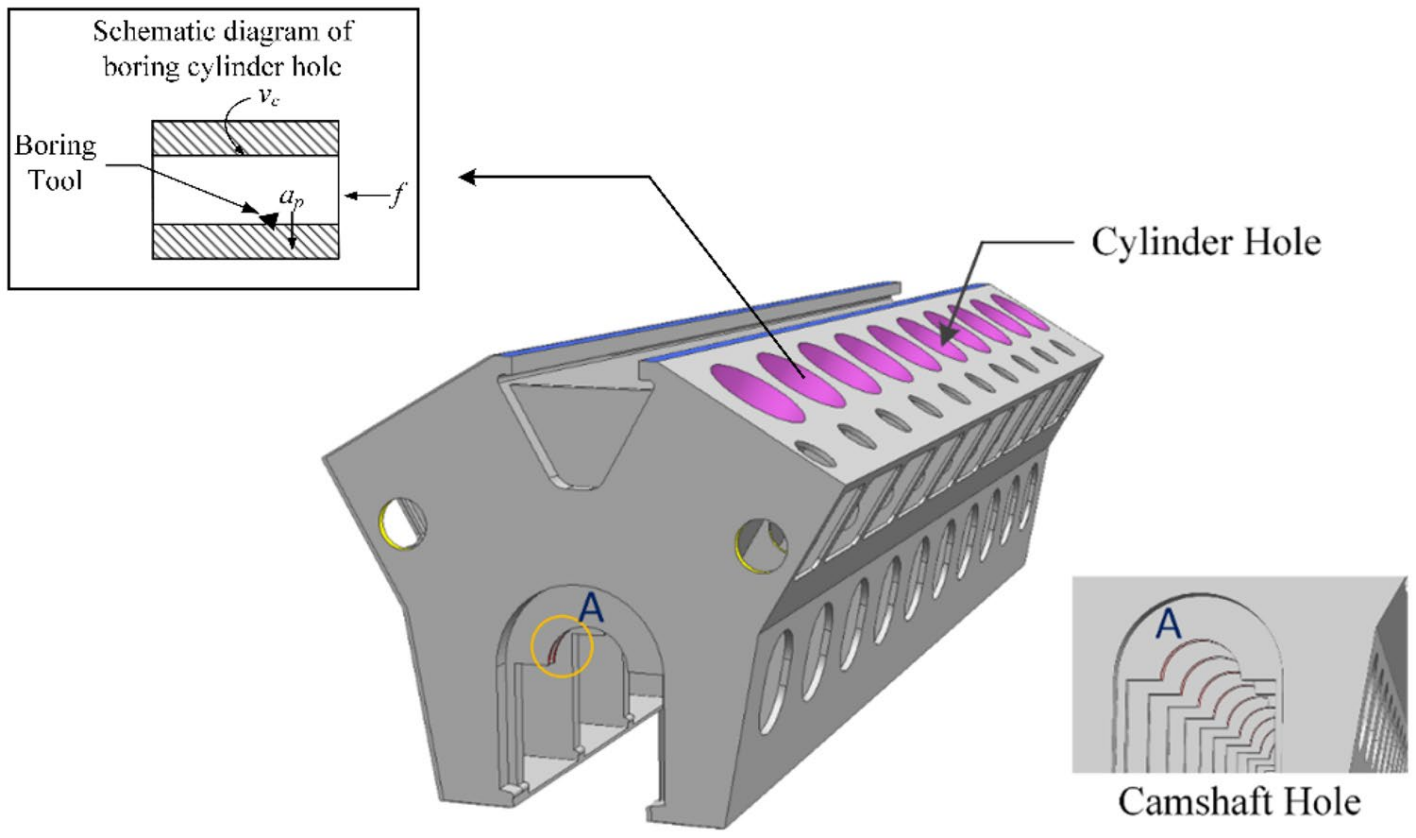
Three-dimensional simplified model of the diesel engine block.

The three-dimensional simplified model of the diesel engine block is shown in Fig. 1. During the operation of a marine diesel engine, the piston and the crankshaft movement center are in a vertical relationship. The verticality of the cylinder hole to the crankshaft hole axis directly affects the reliability of the diesel engine. Therefore, the minimum verticality of the cylinder hole to the crankshaft hole is the optimization goal. Process parameters are closely related to the cutting force and cutting temperature of the diesel engine block. It is easy to cause machining defects such as diesel engine block machining deformation with improper cutting force and cutting temperature. These machining defects in the machining accuracy of diesel engine block will be manifested as out of dimensional tolerance, geometric tolerance, and surface quality, which will directly affect the operation of the transmission shaft and other diesel engine performance problems. Therefore, the process parameters (cutting speed *v*_*c*_, cutting depth *a*_*p*_, and feed rate *f*) are selected as the variables to be optimized. According to the design parameters of the diesel engine block and production experience, the parameter value range setting of design variables is shown in Table 1. *X*_*i*_^(*L*)^and *X*_*i*_^(*U*)^ means the upper and lower limits of design variables.

**Table 1:** Parameter range of design variables.

Design variable	*v*_*c*_(m/min)	*a*_*p*_(mm)	*f*(mm/r)
*X*_*i*_^(*L*)^	99.852	0.3	0.4
*X*_*i*_^(*U*)^	166.42	0.5	0.6

### Experimental design

**Figure 2:**
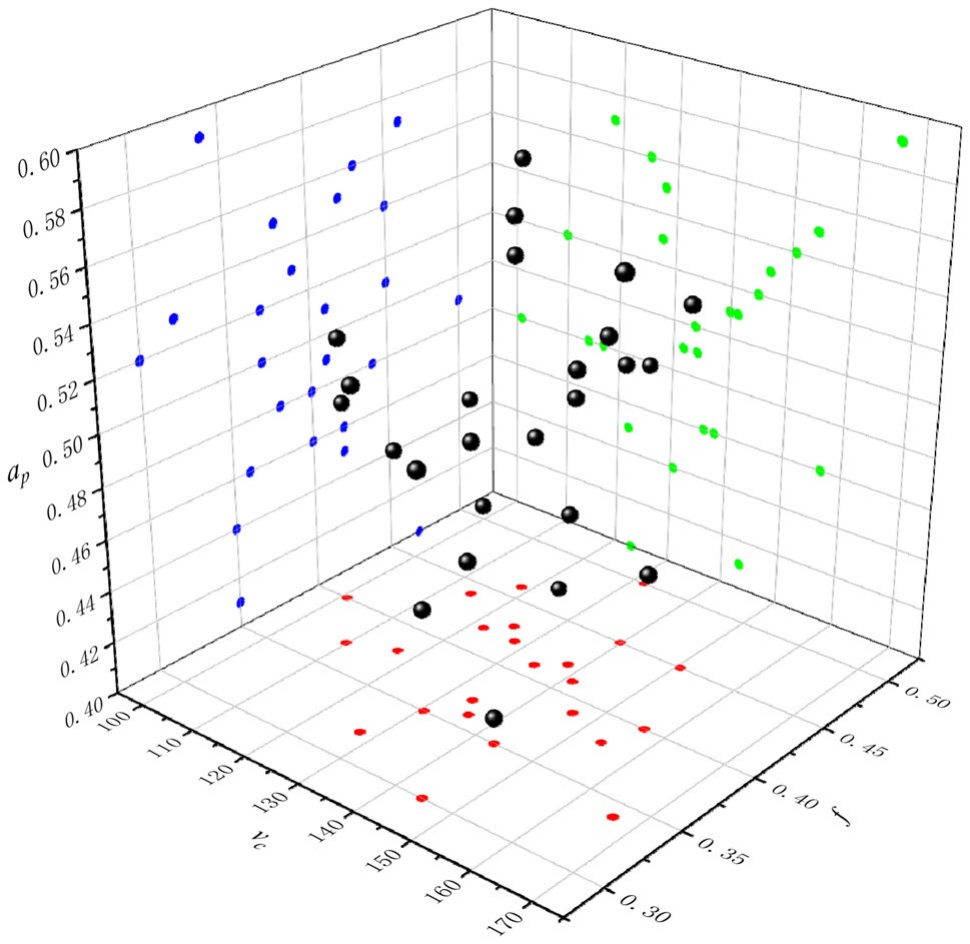
Three-dimensional distribution diagram of sample points of design variables.

The construction of the approximate model depends on the selection of sample points. Commonly used experimental design methods include Design of experimental, Orthogonal experimental design, Central composite design, and Latin hypercube sampling. Among them, the Latin hypercube sampling has good uniformity and projection characteristics, which can make all test points as evenly distributed in the design space as possible, thereby improving the fitting accuracy of factors and responses^16^. Therefore, the Latin hypercube sampling is used to select the three design variables, and their distribution is shown in Fig. 2. In Fig. 2, the black dot represents the collected design variables (*v*_*c*_, *f*, *a*_*p*_) test points by the Latin hypercube sampling, the red dot represents the projection of the process parameter sample points on the *v*_c_-*f* plane, the blue dot represents the projection of the process parameter sample points on the *a*_*p*_-*f* plane, and the green dot represents the projection of the process parameter sample points on the *v*_*c*_-*a*_*p*_ plane.

According to the three-dimensional scatter plot, it can be seen that the distribution of sample points in the design space is relatively uniform, and each area is occupied by sample points, and the space utilization rate is high. Based on the sample points of the Latin hypercube sampling, the cylinder hole machining test was carried out, and the test results are shown in Table 2.

**Table 2:** Sampling test results of Latin hypercube test.

Sample number	*v*_*c*_(m/ min)	*a*_*p*_(mm)	*f*(mm/r)	⊥(mm)
1	156.546	0.4	0.46	0.0503
2	139.838	0.45	0.51	0.0507
3	143.288	0.35	0.41	0.0495
4	122.052	0.42	0.45	0.0469
5	141.231	0.41	0.51	0.0457
6	118.453	0.46	0.57	0.0531
7	109.77	0.37	0.52	0.0439
8	114.028	0.44	0.48	0.0501
9	150.647	0.45	0.54	0.0521
10	130.538	0.35	0.44	0.048
11	116.903	0.38	0.48	0.047
12	146.499	0.39	0.53	0.0508
13	123.032	0.47	0.4	0.0519
14	134.297	0.41	0.49	0.0445
15	166.325	0.34	0.59	0.0535
16	135.912	0.36	0.46	0.0509
17	127.579	0.32	0.53	0.0477
18	137.862	0.42	0.46	0.0478
19	125.124	0.43	0.56	0.0492
20	131.848	0.5	0.49	0.0533
21	154.121	0.38	0.55	0.0478
22	144.647	0.3	0.52	0.0504
23	100.885	0.4	0.48	0.0492
24	133.862	0.37	0.5	0.0492
25	127.921	0.42	0.55	0.0485

### Verticality of cylinder hole to the crankshaft hole modeling based on RBF method

The relationship between the verticality of the cylinder hole to the crankshaft hole and design variables needs to be determined through machining tests. The process of optimizing process parameters involves multiple tests, which results in serious time consumption and cost. Therefore, approximate model technology is used to build a model that meets the accuracy requirements and has a low computational cost to establish the mapping relationship between variables and responses. Subsequent optimization work based on this model will greatly reduce the optimization cost and speed up the optimization process.

Commonly used approximate models include the response surface model, multiple adaptive regression spline model, kriging model, support vector regression model (SVR), and radial basis function model (RBF). Process parameter optimization of the diesel engine block hole system is a small sample problem. In the case of limited sample size, SVR and RBF can obtain better fitting results. Compared with SVR, RBF has a more prominent fitting effect on nonlinear problems^17^, so the RBF method is chosen to establish an approximate model of cylinder hole verticality.

**Figure 3:**
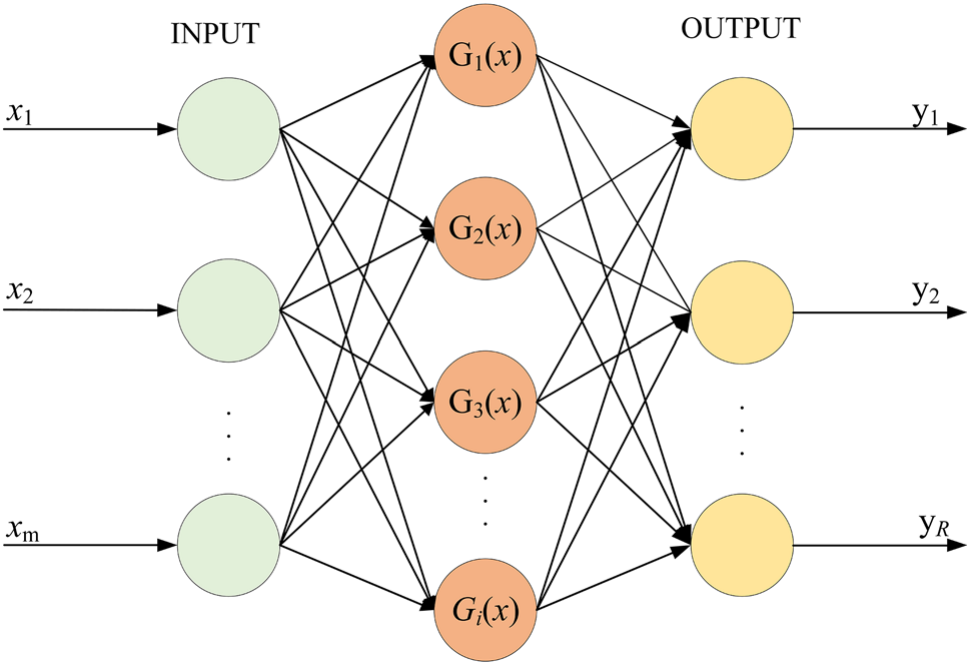
RBF neural network structure diagram.

The basis of the RBF is the function approximation theory, which is a feedforward neural network with strong global optimization capabilities^18^. The RBF is usually composed of an input layer, a hidden radial basis layer, and an output linear layer, the network structure of the RBF is shown in Fig. 3. The radial basis function is radially symmetric, and the Gaussian function is commonly used:

1 $$ G_{i} (x) = \exp ( - \frac{{||x - c_{i} ||^{2} }}{{2\sigma_{i}^{2} }}),i = 1,2, \ldots ,p $$

Among them, *x* is the m-dimensional input vector; *c*_*i*_ is the center of the *i*-th basis function; *σ*_*i*_ is the variance of the *i*-th basis function; *p* is the number of perceptual units.

The input layer of the RBF network realizes the nonlinear mapping from *x* → *G*_*i*_(*x*), and the output layer realizes the linear mapping from *G*_*i*_(*x*) → *y*_*R*_, namely:

2 $$ y_{R} = \sum\limits_{i = 1}^{p} {w_{ki} } G_{i} (x),k = 1,2, \cdots ,q $$

Among them, *q* is the number of output nodes, *w*_*ki*_ is the adjustment weight between the *k*-th output layer and the *i*-th hidden layer nerve.

### RBF model prediction results and analysis

The RBF model is trained through the cylinder hole machining test result data, and the test data with sample number 16–25 in Table 2 is used as the test sample to verify the prediction accuracy of the trained RBF model. The comparison between the actual value of the cylinder hole verticality in the test set and the cylinder hole verticality predicted by the trained RBF model is shown in Table 3.

**Table 3:** Comparison of the actual value of cylinder hole verticality and RBF predictive value.

Sample number	Input (process parameters)			Output (Verticality of cylinder hole)		
	*v*_*c*_ (m/ min)	*a*_*p*_ (mm)	*F* (mm/r)	Actual value	Predictive value	Relative error
16	135.912	0.36	0.46	0.0509	0.04937	3.01%
17	127.579	0.32	0.53	0.0477	0.047751	0.11%
18	137.862	0.42	0.46	0.0478	0.0473	1.05%
19	125.124	0.43	0.56	0.0492	0.04913	0.14%
20	131.848	0.5	0.49	0.0533	0.053349	0.09%
21	154.121	0.38	0.55	0.0478	0.04773	0.15%
22	144.647	0.3	0.52	0.0504	0.05043	0.06%
23	100.885	0.4	0.48	0.0492	0.049367	0.34%
24	133.862	0.37	0.5	0.0492	0.04887	0.67%
25	127.921	0.42	0.55	0.0485	0.04847	0.06%

R-squared is used to judge the fit of the model, the mathematical model is expressed as follows:

3 $$ R^{2} = 1 - \frac{{\sum\limits_{n = 1}^{m} {(y_{n} - \widehat{y}_{n} )^{2} } }}{{\sum\limits_{n = 1}^{m} {(y_{n} - \overline{y} )^{2} } }} $$

Among them, *m* is the number of Validation sample points, *y*_*n*_ is the actual value of the sample points,$\widehat{y}_{n}$ is the predicted value calculated by the approximate model, and $\overline{y}$ is the average value of the test sample point set. When the *R*^2^ value is closer to 1, the accuracy of the approximate model is higher.

The root mean square error is used to measure the deviation between the predicted value and the actual value. The expression is as follows:

4 $$ RMSE = \sqrt {\frac{{\sum\limits_{n = 1}^{m} {(y_{n} - \widehat{y}_{n} )^{{2}} } }}{m}} $$

Among them, *m* is the number of test sample points, *y*_*n*_ is the actual value of the test sample points, and $\widehat{y}_{n}$ is the predicted value of the approximate model. The closer the RMSE value is to 0, the smaller the deviation between the predicted value and the actual value.

After calculation, the R-square value corresponding to the test sample is 0.904513, and the RMSE value is 0.00005. The established RBF model is relatively accurate and meets the accuracy requirements of the approximate model.

## Establishment of constraints

The residual stress will affect the fatigue strength and life of the diesel engine block, and its release process will deform the cylinder hole of the block, thereby affecting the accuracy and stability of the block. In order to ensure the accuracy of the diesel engine block hole system, the surface residual stress of the diesel engine block after machining is required to be less than a certain value, and the probability that the residual stress after machining is less than a given value is used as the reliability index.

**Figure 4:**
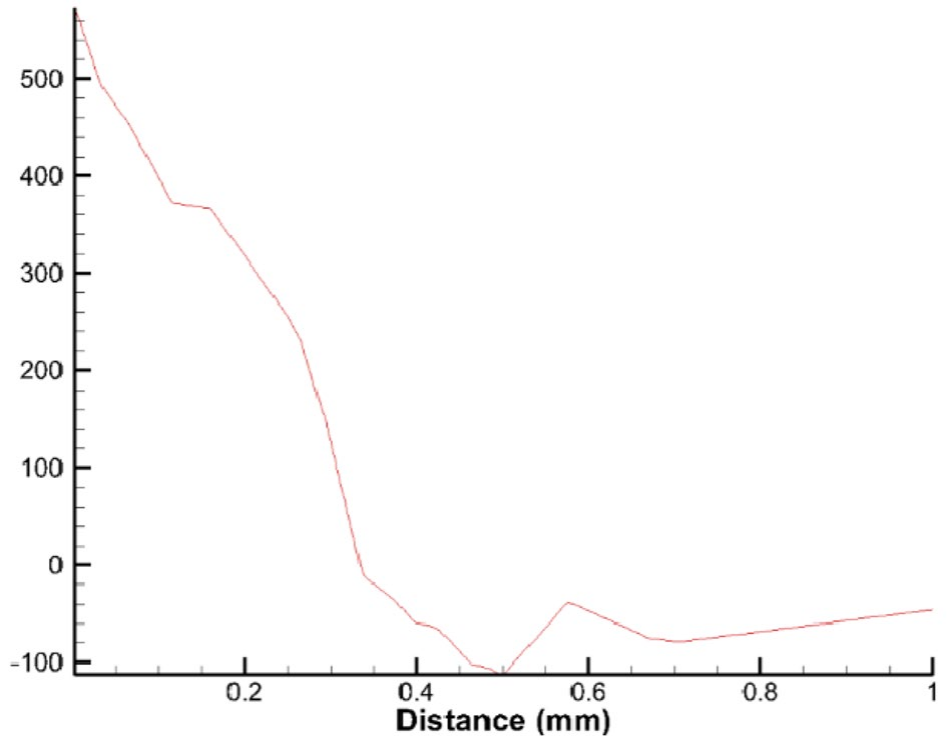
The curve of residual stress with depth distance output by the Third Wave AdvantEdge FEM 7.1.

The Third Wave AdvantEdge FEM 7.1 software^19^ is used to simulate the residual stress after the cylinder hole boring. Figure 4 is the curve of the residual stress along with the depth distance after the cylinder hole boring output by the Third Wave AdvantEdge FEM 7.1 software post-processing module, when the cutting speed is 166.46r/min, the cutting depth is 0.4 mm, and the feed rate is 0.4 mm/r. According to Fig. 4 output by the Third Wave AdvantEdge FEM 7.1, it can be seen that the residual stress on the surface of the workpiece is mainly tensile stress, and the residual stress in the inner layer of the workpiece is mainly compressive stress; the surface residual stress is the largest, and the residual tensile stress decreases rapidly along with the depth of the layer. The residual stress transitions to compressive stress at a depth of about 0.3 mm. The compressive residual stress reaches its maximum at a depth of 0.5 mm, and then the residual stress slowly tends to 0.

Cutting speed, cutting depth, and feed rate are taken as independent variables, and the residual stress on the cylinder hole surface obtained by the Third Wave AdvantEdge FEM 7.1 software simulation is the response value. According to the central composite response surface method test plan, 17 sets of residual stress simulation tests by the Third Wave AdvantEdge FEM 7.1 are carried out. The test results are shown in Table 4.

**Table 4:** Simulation test results of residual stress obtained by the Third Wave AdvantEdge FEM 7.1.

Test number	Coding variable			Actual variable			Response
	*X*_1_	*X*_2_	*X*_3_	*v*_*c*_	*a*_*p*_	*f*	*σ*_*R*_ (Mpa)
1	− 1	1	0	99.852	0.5	0.5	270
2	1	− 1	0	166.46	0.3	0.5	466
3	0	1	− 1	133.156	0.5	0.4	600
4	0	1	1	133.156	0.5	0.6	642
5	− 1	− 1	0	99.852	0.3	0.5	390
6	0	− 1	1	133.156	0.3	0.6	575
7	− 1	0	− 1	99.852	0.4	0.4	270
8	0	0	0	133.156	0.4	0.5	510
9	1	0	− 1	166.46	0.4	0.4	565
10	0	− 1	− 1	133.156	0.3	0.4	523
11	0	0	0	133.156	0.4	0.5	510
12	− 1	0	1	99.852	0.4	0.6	315
13	1	0	1	166.46	0.4	0.6	530
14	0	0	0	133.156	0.4	0.5	510
15	0	0	0	133.156	0.4	0.5	510
16	1	1	0	166.46	0.5	0.5	517
17	0	0	0	133.156	0.4	0.5	510

Based on the test data, the response surface fitting model is established as shown in (5).

5 $$ \begin{gathered} \sigma_{R} = - 308 + 32.72 \times v_{c} - 4100 \times a_{p} - 3200 \times f - \hfill \\ 0.12 \times v_{c}^{2} + 3300 \times a_{p}^{2} + 4200 \times f^{2} + 12.84 \times v_{c} \times a_{p} - \hfill \\ 6.01 \times v_{c} \times f - 250 \times a_{p} \times f \hfill \\ \end{gathered} $$

**Figure 5:**
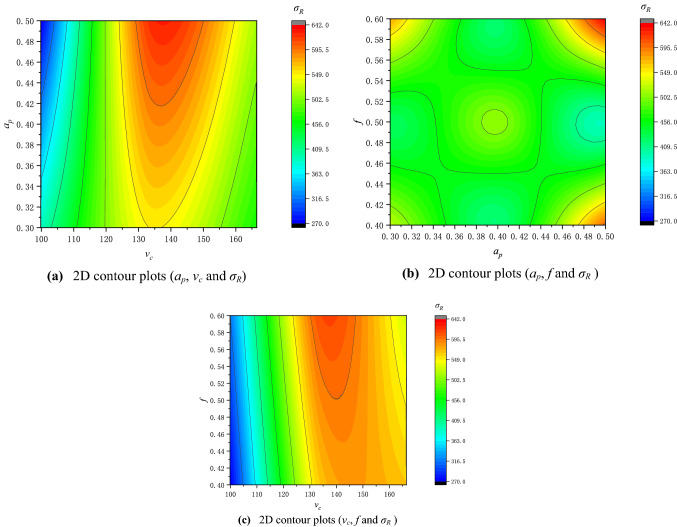
2D contour plots of the process parameters.

According to the response surface model, the contour plot between the process parameters is drawn, as shown in Fig. 5.

It can be seen from the contour diagram that the cutting speed has the greatest influence on the residual stress on the surface of the cylinder hole, followed by the cutting depth and the feed rate, and the larger residual stress is concentrated at the cutting speed of 130r/min-155r/min.

It is assumed that the process parameters meet the normal distribution, and the mean and standard deviation are set as shown in Table 5, *N* (*μ*, *σ*) represents a normal distribution with the mean value of *μ* and standard deviation of *σ*.

**Table 5:** The distribution parameters of machining parameters.

Distribution	*v*_c_	*a*_*p*_	*f*
*N* (*μ*, *σ*)	*N* (99.852,10)	*N* (0.4,0.01)	*N* (0.5,0.02)

The Monte Carlo method (MCM) is a numerical calculation method that generates random numbers based on the probability distribution of the input and realizes distribution propagation by re-sampling them.

**Figure 6:**
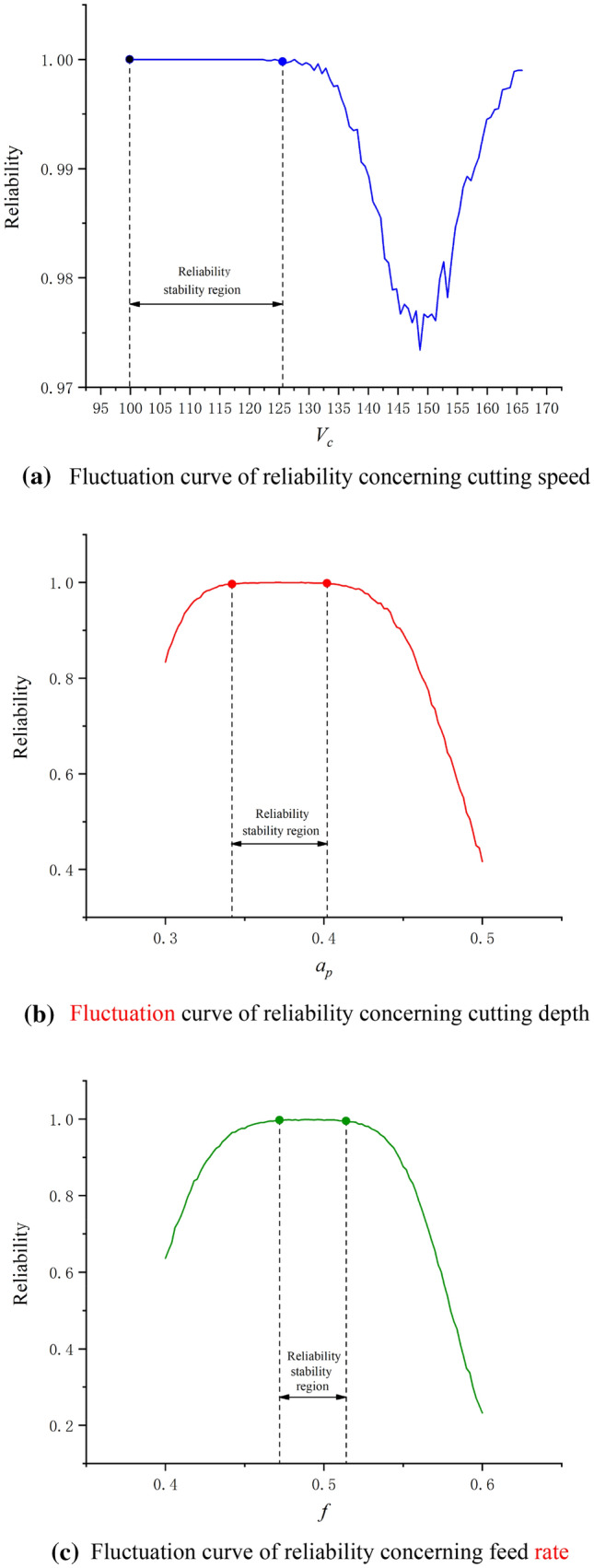
Reliability fluctuation curve.

Based on the established response surface model, MCM is used to perform 10,000 calculations to count the number of unsatisfactory residual stresses on the surface, so that the process reliability of the cylinder hole can be obtained. The allowable value of surface residual stress is set to 550Mpa. The standard deviations of the three variables remain unchanged and change the variable mean to calculate the corresponding reliability. The fluctuation curves of the reliability concerning the three variables are shown in Fig. 6.

According to the fluctuation curve, it can be seen that the influence of cutting speed on block reliability is not significant, but the cutting depth and feed rate have a great influence on block reliability, and their fluctuation curves are similar. It is speculated that both cutting depth and feed rate are related to the material cutting amount. When the cutting depth and feed rate are small, the chip takes away a lot of heat, which reduces the residual stress of the cylinder hole, With the increase of cutting speed and feed rate, the heat carried by the chip is limited, and the cutting thermal effect is enhanced, which leads to the continuous increase of residual stress on the surface of the cylinder hole the variable interval with relatively stable reliability fluctuation is selected as the reliability stability region constraint of the diesel engine block cylinder hole machining, as shown in Table 6, where *X*_*R*_^(*L*)^ and *X*_*R*_^(*U*)^ respectively represent the reliability stability region interval the lower limit and upper limit.

**Table 6:** Reliability stability region.

Design variable	*v*_*c*_(m/min)	*a*_*p*_(mm)	*f*(mm/r)
*X*_*R*_^(*L*)^	99.852	0.342	0.472
*X*_*R*_^(*U*)^	124.932	0.402	0.514

## Reliability optimization of process parameters

### Process parameters optimization model

The main research in this paper is to minimize the verticality of the cylinder bore by optimizing the process parameters. Based on objective function and the reliability stability region constraints, an optimization model of the cylinder hole process parameters of the diesel engines block can be constructed:

Find *v*_*c*_, *a*_*p*_, *f*

Min *V*(*v*_*c*_, *a*_*p*_, *f*).

99.852 m/min ≤ *v*_*c*_ ≤ 124.932 m/min

0.342 mm ≤ *a*_*p*_ ≤ 0.402 mm.

0.472 mm/r ≤ *f* ≤ 0.514 mm/r.

Among them, *v*_*c*_, *a*_*p*_, *f* are the cutting speed, depth of cut, and feed rate, respectively, and *V* is the verticality of the cylinder hole to the crankshaft hole.

### Particle swarm single-objective optimization algorithm based on hooke-jeeves algorithm

#### Hooke-Jeeves algorithm description.

Hooke-Jeeves is a direct search method. Its core idea is to find out the optimal descent direction of the function by calculating and comparing the value of the function to solve the target optimization problem^20^. The search steps are as follows:

Step1: The initial point *x*^(1)^ ϵ *R*^n^ and initial step size *δ* is given, acceleration factor *α* ≥ 1, reduction rate *β* ϵ (0,1), accuracy *ε* > 0. *y*^(1)^ = *x*^(1)^, *k* = 1, *j* = 1 are set;

Step2: If *f*(*y*^(*j*)^ + *δe*_*j*_) < *f*(*y*^(*j*)^), then *y*^(*j*+*1*)^ = *y*^(*j*)^ + *δe*_*j*_, and Step4 is going to be executed; otherwise, Step 3 is going to be executed;

Step3: If *f*(*y*^(*j*)^-*δe*_*j*_) < *f*(*y*^(*j*)^), then *y*^(*j*+*1*)^ = *y*^(*j*)^-*δe*_*j*_, and Step4 is going to be executed; otherwise, *y*^(*j*+1)^ = *y*^(*j*)^, step 4 is going to be executed;

Step4: If *j* < *n*, then *j*: = *j* + 1, step 2 is going to be executed; otherwise, Step5 is going to be executed;

Step5: If *f*(*y*^(*n*+1)^) < *f(x*^(*k*)^), then Step6 is going to be executed; otherwise, Step7 is going to be executed;

Step6: *x*^(*k*+1)^ = *y*^(*n*+1)^, *y*^(1)^ = *x*^(*k*+1)^ + *α*(*x*^(*k*+1)^-*x*^(*k*)^), *k*: = *k* + 1, and *j* = 1,then Step2 is going to be executed;

Step7: If *δ* ≤ *ε*, the iteration should stop and the point *x*^(*k*)^ is get. Otherwise, *δ*: = *βδ*, *y*^(1)^ = *x*^(*k*)^, *x*^(*k*+1)^ = *x*^(*k*)^, *k*: = *k* + 1, and *j* = 1, then Step2 is going to be executed.

According to the search step of the Hooke-Jeeves method, it can be seen that the search efficiency is greatly affected by the position of the initial point. For different initial points, the optimization accuracy and optimization speed will fluctuate greatly. Therefore, in order to ensure that it can efficiently search for the best, we should ensure that it has a better initial position.

#### Improved particle swarm optimization algorithm based on hooke-jeeves algorithm

The particle swarm optimization algorithm (PSO) is derived from the study of bird predation behavior. It uses a particle to simulate individual birds. Each particle is regarded as a searching individual in the search space. The current position of the particle is a candidate solution for the optimization problem. The flight process is the process of searching for individuals. Particles have two attributes: speed and position. Speed represents the speed of movement, and position represents the direction of movement. The optimal solution searched for by each particle is called the individual extreme value. The optimal individual extremum in the particle swarm is the global optimal solution to the optimization problem that is sought. The speed and position are constantly updated to iterate, and finally, the optimal solution that reaches the termination condition is obtained. The calculation formula for the update speed V_*id*_ and position X_*id*_ is as follows:

6 $$ V_{id} = \omega V_{id} + C_{1} rand()(P_{id} - X_{id} ) + C_{2} rand()(P_{gd} - X_{id} ) $$

7 $$ X_{id} = X_{id} + V_{id} $$

where *ω* is the inertia factor, *C*_1_ and *C*_2_ are the individual learning factor and the environmental learning factor respectively, and their value range is [0,4] generally, *rand*() is the random number on [0,1], *P*_*id*_ represents the *d*-th dimension of the individual extremum of the *i*-th variable and *P*_*gd*_ represents the *d*-th dimension of the global optimal solution.

**Figure 7:**
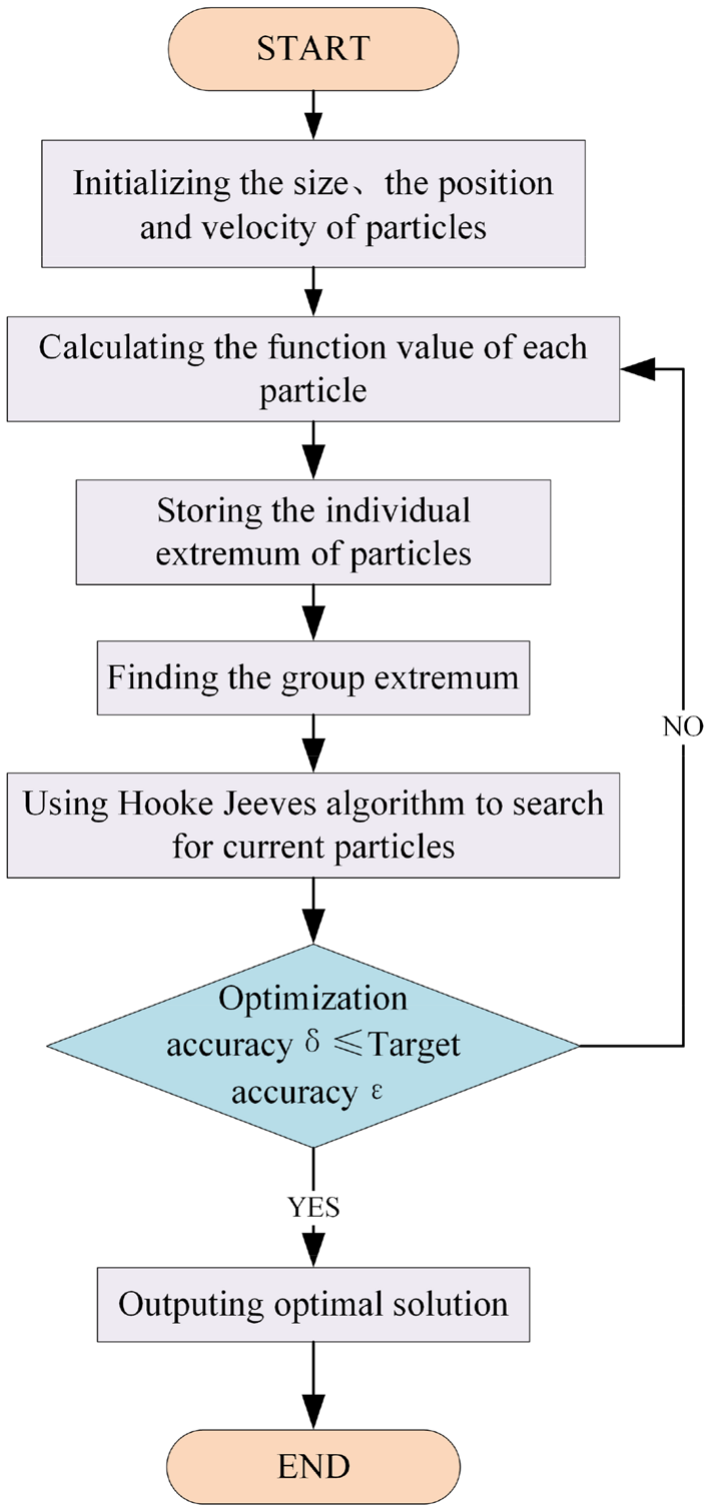
Optimization step diagram of improved particle swarm algorithm based on Hooke-Jeeves algorithm.

The flying speed of the particles in the particle swarm optimization algorithm affects the global convergence of the algorithm. A larger speed can ensure that the particles quickly fly to the vicinity of the optimal solution, but they will fall into the dilemma of local optimality^21^. Therefore, this paper combines the Hooke-Jeeves algorithm with the particle swarm optimization algorithm. First, the particle swarm optimization algorithm is used to locate the area where the target extremum is located in the design space, and then the Hooke-Jeeves algorithm is used to accurately search the area, and finally, the best design result is obtained. The steps are shown in Fig. 7.

### Algorithm parameter setting

In order to prove the superiority of the improved particle swarm optimization algorithm based on the Hooke-Jeeves algorithm (HJ-PSO) in solving parameter optimization problems, the algorithm is compared with the particle swarm algorithm. The particle swarm optimization algorithm parameters are set as: inertia factor *ω* = 0.9, individual learning factor, and environmental learning factor *C*_1_ = *C*_2_ = 0.9, the number of max iterations is 20, the number of particles is 10, and the maximum flight speed is 100. The parameters of HJ-PSO are set as follows: initial step size *δ* = 0.5, reduction rate *β* = 0.5, acceleration factor *α* = 1, accuracy *ε* = 10^–6^, other parameters are the same as the particle swarm algorithm, and its parameter settings are shown in Table 7.

**Table 7:** HJ− PSO algorithm parameters.

Maximum iterations of PSO	10
Maximum iterations of Hooke–Jeeves	10
Inertia factor *ω*	0.9
Individual learning factor *C*_1_	0.9
Environmental learning factor *C*_2_	0.9
Number of particles *M*	10
Maximum flight speed *V*_max_	100
Initial step *δ*	0.5
Reduction rate *β*	0.5
Acceleration factor *α*	1
Accuracy *ε*	10^–6^

### Analysis of optimization results

PSO and HJ-PSO is used to solve the optimization model respectively. All the algorithms run independently 20 times, and the average value and variance of the objective function optimization results of each algorithm are shown in Table 8.

**Table 8:** Comparison of optimization results.

	PSO	HJ-PSO
Mean value	0.04336	0.04302
Standard deviation	5.262E-4	4.956E-5

According to the mean comparison of the optimization results, it can be seen that under the same number of iterations, HJ-PSO gets better optimization results than PSO algorithm when searching the global optimal value. In addition, the comparison of standard deviation shows that the stability of HJ-PSO is significantly better than that of PSO.

**Figure 8:**
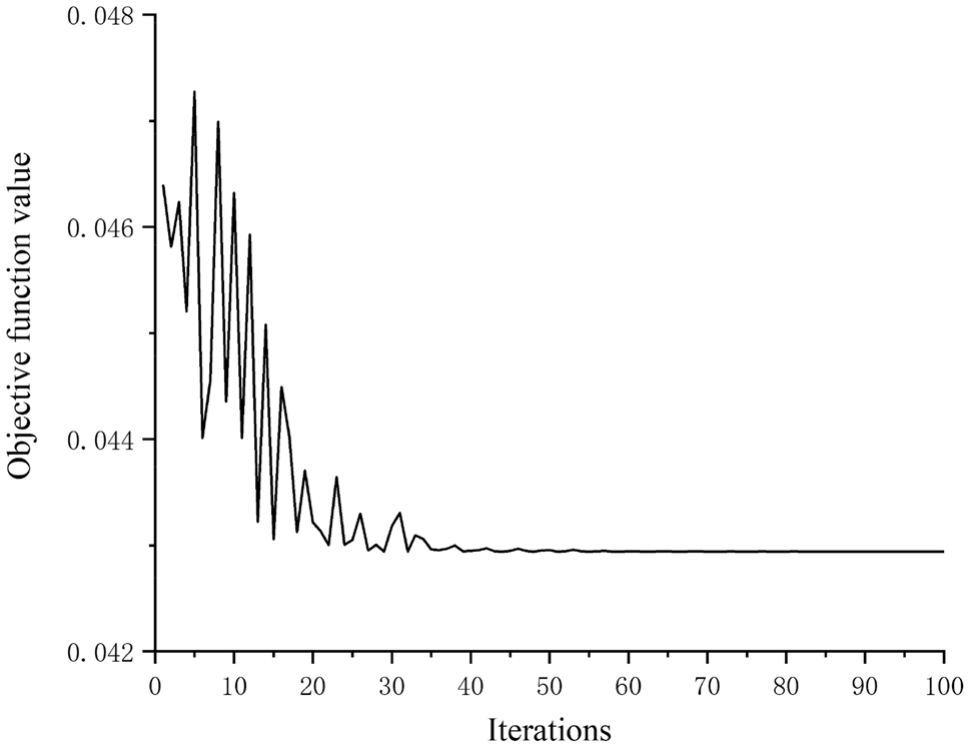
Solving process of HJ-PSO.

The number of iterations is set to 100, and HJ-PSO is used to solve the optimal combination of process parameters for the verticality machining of the cylinder hole. The solving process is shown in Fig. 8.

As can be seen from the figure that the HJ-PSO algorithm converges when the number of iterations reaches about 35, and the corresponding objective function value is 0.04294.

### Verification test

**Figure 9:**
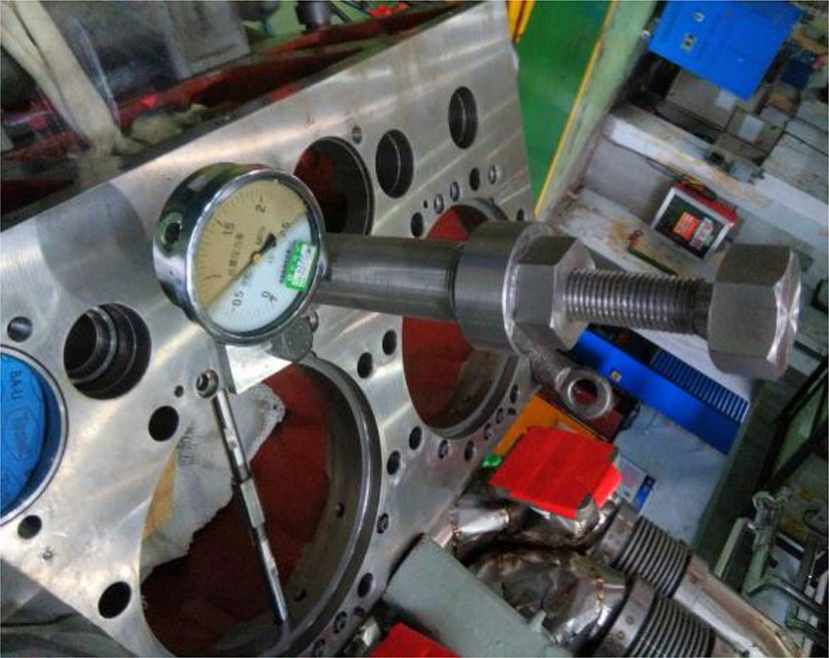
Cylinder hole of the workpiece.

The optimal combination of process parameters can be obtained by improving the particle swarm algorithm as follows: cutting speed is 99.852 m/min, cutting depth is 0.352 mm, and feed is 0.508 mm/r. The obtained combination of process parameters is used to perform a verification test. The verticality of the cylinder hole measured by the verticality measuring instrument is 0.0436, which is less than the minimum verticality in the historical data. The cylinder hole of the workpiece is shown in Fig. 9. However, the error between the optimization result value and true value is 1.53%, and it is considered as the influence of process system error.

## Conclusions

In this paper, based on the reliability theory, the improved particle swarm optimization algorithm is used to optimize the processing parameters of the cylinder hole of the diesel engine block. The optimal combination of process parameters obtained based on this method can guide the process engineer to improve the process of the diesel engine block or other similar products. As follows are the important conclusions in this paper.

1. The cutting speed has the greatest influence on the surface residual stress of the cylinder hole, followed by the cutting depth and feed rate.
2. The reliability of the cylinder hole machining process fluctuates greatly with the change of cutting depth and feed rate, less with the change of cutting speed.
3. Compared with the general particle swarm optimization algorithm, the efficiency and results of the improved particle swarm optimization algorithm are improved.
4. Through the improved particle swarm optimization algorithm based on the Hooke-Jeeves algorithm, the optimal combination of processing parameters of cylinder hole is obtained as follows: the cutting speed is 99.852 m/min, the cutting depth is 0.352 mm, the feed rate is 0.508 mm/r. Based on the optimal combination of processing parameters of the cylinder hole, the actual processing of the block can be guided.

Compared with Reference^15^, which only evaluates the process reliability of diesel engine block, this paper not only expounds on the evaluation of process reliability of diesel engine block, but also obtains the optimal combination of process parameters that meet the requirements of process reliability through optimization algorithm, which can guide the production of diesel engine block more effectively. However, the process of the diesel engine block is complex, so the study in this paper only focuses on the process parameters of the process. In the future, the quality transfer relationship between the multiple processes of the block can be studied, and more accurate optimization results can be obtained.
